# Elevated serum uric acid is associated with the risk of advanced staging and vascular involvement in patients with hepatoblastoma: a 14-year retrospective study

**DOI:** 10.3389/fonc.2023.1144349

**Published:** 2023-04-14

**Authors:** Yunlan Zhou, Jinning Li, Yanhui Ma, Mengjie Tang, Xiaojun Yuan, Lisong Shen

**Affiliations:** ^1^Department of Clinical Laboratory, Xinhua Hospital, Shanghai Jiao Tong University School of Medicine, Shanghai, China; ^2^Faculty of Medical Laboratory Science, College of Health Science and Technology, Shanghai Jiao Tong University School of Medicine, Shanghai, China; ^3^Institute of Artificial Intelligence Medicine, Shanghai Academy of Experimental Medicine, Shanghai, China; ^4^Department of Radiology, Xinhua Hospital, Shanghai Jiao Tong University School of Medicine, Shanghai, China; ^5^Department of Pediatric Hematology/Oncology, Xinhua Hospital, Shanghai Jiao Tong University School of Medicine, Shanghai, China

**Keywords:** hepatoblastoma, serum uric acid, PRETEXT, staging, imaging, risk evaluation

## Abstract

**Background:**

Uric acid is the end product of the purine metabolism pathway, and has been linked to cancer risks and prognosis, but its relationship with hepatoblastoma (HB) remains unclear. This study aims to investigate the association between serum uric acid (SUA) and the advanced tumor staging and unfavorable extra-parenchymal tumor characteristics in patients with HB.

**Methods:**

This study enrolled pediatric patients from Xinhua Hospital between 2007 to 2021. A total of 101 participants with newly diagnosed HB were recruited in the study. PRETreatment EXTent of disease (PRETEXT)/PostTreatment Extent of disease (POSTTEXT) staging were evaluated at diagnosis and following neoadjuvant chemotherapy (NAC). Adjusted smoothing spline plots, subgroup analysis and multivariate logistic regression analysis were conducted to estimate the association of different levels of SUA with the advanced tumor staging and present annotation factors.

**Results:**

In accordance with SUA tertiles, those patients with higher pretreatment SUA levels showed increased percentages of PRETEXT group IV, vessel involvement and multifocality of tumors. After fully adjustment with the confounding factors, SUA was positively associated with advanced PRETEXT stage IV (OR: 1.72, 95%CI 1.15-2.57, *p*=0.0080), as well as vascular invasion (OR: 1.29, 95%CI 1.01-1.64, *p*=0.0396). Compared with the lowest SUA concentration tertile, the highest tertile were independently associated with vessel involvement of tumor in all of the adjusted models. Following NAC, SUA levels were significantly reduced in response to the downstaging of tumors. SUA remained positively associated with advanced POSTTEXT staging and vessel involvement in adjusted models. Patients with highest tertile of posttreatment SUA showed worse 5-year EFS and OS.

**Conclusion:**

Elevated SUA were associated with an increased occurrence of advanced PRETEXT/POSTTEXT staging and unfavorable vessel involvement at diagnosis and following NAC in patients with HB. High posttreatment SUA reflected poor tumor responses to NAC. This study linked SUA, a non-invasive laboratory test, with tumor staging and risk prediction for HB.

## 1 Introduction

Hepatoblastoma (HB) is the predominant liver tumor in children with an annual incidence of 1.5 per million approximately (1). Treatments combining chemotherapy and surgical resection have greatly improved the outcomes for HB. Nevertheless, challenges remain for the treatment options in clinically advanced tumors (2, 3). PRETreatment EXTent of disease (PRETEXT) system is the most widely used HB staging system for disease evaluation and risk stratification basing on the number of liver sectors that are invaded by tumor on radiographic imaging (4). The newly updated PRETEXT system standardizes the annotation factors describing features including vascular involvement, extrahepatic disease, multifocality, tumor rupture and metastases (5, 6). Patients with PRETEXT staging IV and unfavorable annotation features have been related to worse overall survival and event-free survival (7). Following neoadjuvant chemotherapy (NAC), tumor extension are again assessed radiologically to evaluate resectability, which result in the PostTreatment Extent of disease (POSTTEXT) staging. Thus, routine clinical laboratory test associated with PRETEXT/POSTTEXT staging may act as a useful marker for disease evaluation and provide practical information to the clinical care team.

Uric acid is the end product of the purine metabolism pathway, and is an antioxidant scavenging free radicals in the body (8, 9). Recent evidences have linked serum uric acid (SUA) to cancer risks in adulthood (10, 11). Studies show that hyperuricemia is associated with an increased cancer incidence and mortality (12), while some other conflicting reports have suggested a protective role of SUA in cancer due to its antioxidant properties (13, 14). However, the relationship of SUA with pediatric solid tumors is largely unknown. Given that uric acid is mainly produced in the liver from degradation of dietary and endogenous purine compounds, there has been growing interest in the role of SUA playing in the development of liver cancer. Recent report reveals that high SUA could be a significant risk factor of adult liver malignancy, suggesting that SUA contributes to the hepato-carcinogenesis (15). To date, studies investigating the association between SUA and pediatric liver tumor are still scarce.

Therefore, this study aims to explore the relationship between SUA and HB, focusing on the disease staging and extra-parenchymal features such as vessel involvement that are critical for treatment selection and patient prognosis.

## 2 Materials and methods

### 2.1 Study population

All HB patients diagnosed in Xinhua Hospital Affiliated to Shanghai Jiao Tong University School of Medicine from January 2007 to December 2021 were eligible for the retrospective analysis. The study was approved by the Ethics Committee of Xinhua Hospital. All patients provided informed consent. The inclusion criteria included: 1) newly diagnosed patients; 2) age < 18 years; 3) pathological confirmation of HB by biopsy or surgical resection; 4) pretreatment and posttreatment chest and abdominal computed tomography (CT) or magnetic resonance imaging (MRI) available; 5) laboratory measurements provided prior to and after treatment.

**Figure 1:**
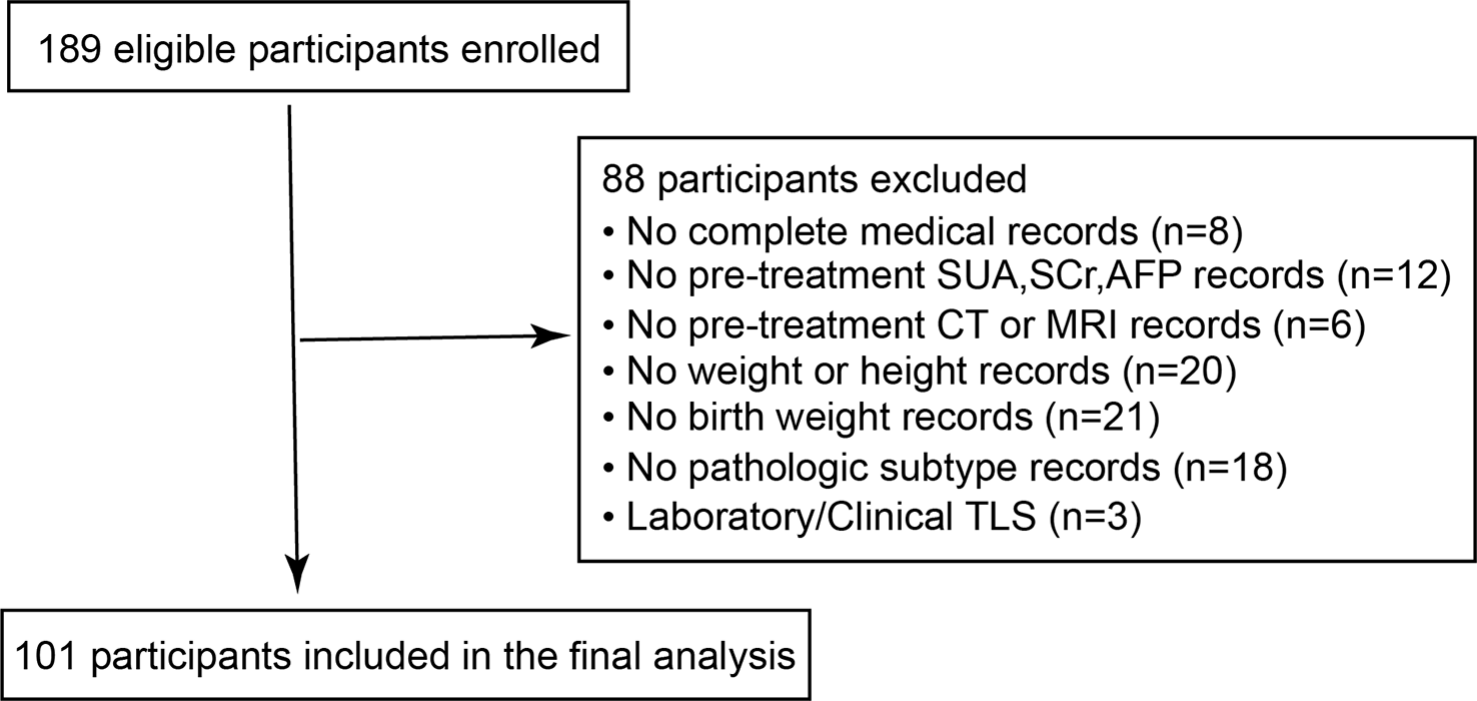
Flowchart of participants inclusion and exclusion.

Clinical characteristics were collected *via* medical records including age at diagnosis, sex, weight, height, birth weight. Body mass index (BMI) was calculated as weight divided by height squared (kg/m^2^). Children with laboratory/clinical tumor lysis syndrome (TLS) were not included in the study (n=3). Laboratory spontaneous TLS was defined as the presence of ≥2 of the hyperuricemia, hyperkalemia, hyperphosphatemia and hypocalcemia prior to administration of chemotherapy (16). We also excluded subjects with missing information on SUA, serum alpha-fetoprotein (AFP), serum creatinine (SCr) (n=12) and other clinical records (n=73). Eventually, a total of 101 HB patients were included in the present analysis **(** **Figure 1****)**.

Data about the radiologic assessments and laboratory measurements were collected both at the time of initial diagnosis and the end of 4th cycle of neoadjuvant chemotherapy(NAC). For those who underwent surgery before 4 cycles of NAC, information on 2^nd^ cycle were used. Five patients had surgical resection at diagnosis without chemotherapy, based on the risk evaluation of their disease.

### 2.2 PRETEXT and POSTTEXT staging

Radiologic evaluations were performed at diagnosis and post-NAC by PRETEXT/POSTTEXT groups and annotation factors. All HB patients were re-evaluated for tumor grades and potential prognostic factors in accordance with the recently updated 2017 PRETEXT risk-stratified staging system (7). Patients were classified into advanced PRETEXT group (PRETEXT IV) and non-advanced group (PRETEXT I, II, III) which reflected hepatic parenchymal tumor involvement (6). PRETEXT annotation factors elucidated extra parenchymal tumor characteristics including involvement of hepatic vein or inferior vena cava (V), portal bifurcation (P), contiguous extrahepatic tumor (E), multifocality (F), spontaneous tumor rupture (R), caudate lobe involvement (C), lymph node metastasis (N), and distant metastasis (M) (6). In each of the annotation criteria, patients were classified according to the presence or absence of the annotation factor. Based on pretreatment and post-chemotherapy CT or MRI images, PRETEXT/POSTTEXT stage and annotation factors were judged by two experienced pediatric radiologists by consensus.

### 2.3 Laboratory measurements

Blood samples were collected from patients after overnight fast, and were obtained before initial treatment and after indicated treatment cycles. SUA and SCr were determined by enzymatic assay using Hitachi-7600 (Hitachi, Japan). The SUA concentrations expressed in μmol/L was converted to the values expressed in mg/dL to make them comparable to other researches (1 mg/dL = 59.5μmol/L). Serum AFP were determined using automatic analyzer Cobas e601(Roche Diagnostics, Germany) according to the manufacturer’s protocols.

Hyperuricemia was unanimously defined based on sex-specific values in adults (17). But in pediatric subjects, the normal reference range of SUA values differ from report to report. However, most evidences show that SUA levels are similar between boys and girls before puberty (18). In this study, we used the pediatric reference ranges as follows: 80-320μmol/L (1.34-5.38 mg/dL) for age under 10 years, 160-470 μmol/L (2.69-7.90mg/dL) for boys aged 11-15 years, and 140-350 μmol/L (2.35-5.88mg/dL) for girls aged 11-15 years (19). SUA levels were categorized into tertiles.

Serum AFP concentration was classified into four categories (≤100ng/mL, 101–1000 ng/mL, 1001–10^6^ ng/mL, and≥10^6^ ng/mL) according to previous studies (5).

### 2.4 Pathologic subtypes

Histologic subtypes documented in this study were judged based on the diagnostic tumor biopsy prior to chemotherapy. In accordance with World Health Organization histologic and morphologic classification criteria, patients were fit into fetal, embryonal, small-cell undifferentiated, macrotrabecular, cholangioblastic, teratoid and stromal derivatives subtypes (20). The histologic subtypes were confirmed by two senior pathologists.

### 2.5 Patient follow-ups

Patients were followed up at clinic and *via* phone calls. Patient outcomes were assessed by event-free-survival (EFS) and overall survival (OS). EFS was defined as the interval between the date of diagnosis and first relapse, progression, or death, whichever occurred first. OS was defined as the interval between the date of diagnosis and death from any cause. Those without any event were censored at the time of last follow-up (December 31^st^, 2022).

### 2.6 Statistical analysis

For non-normal distribution clinical data, continuous variables were presented as median with interquartile range, and categorical variables were presented as counts with percentages. Kruskal-Wallis test was used to compare continuous data among SUA tertile groups. Categorical variables were analyzed by Chi-Square test, and Fisher’s exact test was used when more than 20% of expected counts were less than 5.

Multiple binary logistic regression analyses were performed to assess the independent relationship between SUA and various outcomes for HB patients, including advanced PRETEXT/POSTTEXT staging and positive annotations.

Adjusted smoothing spline plots of SUA were performed to study the association of SUA with the incidence of advanced PRETEXT staging and positive annotation factors. Subgroup analysis in the logistic regression model examined the relationship between SUA and PRETEXT staging stratified by sex, age, AFP. Test for interaction was used to compare odds ratios between the analyzed subgroups.

The multivariable regression models with adjustment for possible confounding factors were implemented. Crude and adjusted odds ratios (ORs) with 95% confidence intervals (CIs) were analyzed. Furthermore, the association between SUA concentration tertiles and various PRETEXT/POSTTEXT outcomes were evaluated with the lowest tertile as the reference.

Kaplan-Meier method were conducted to generate survival curves. The log-rank test was performed to compare EFS and OS curves. Data were analyzed with the statistical packages R (The R Foundation; http://www.r-project.org; version 3.4.3). All statistical tests were two-sided, and *p*-value <0.05 was considered statistically significant.

## 3 Results

### 3.1 Clinical characteristics of HB participants according to SUA tertiles

This study consisted of 101 pediatric HB participants with 63 males and 38 females enrolled. The median age at the initial diagnosis was 2.15 years (range 0.25-10 years). It has been proved that SUA levels are similar between boys and girls before 10 years old (17, 18, 21, 22). Hence, gender-specific reference intervals were not used in our study because of the age range in pediatric participants.

The general clinical characteristics in HB patients at the time of initial diagnosis were presented according to SUA tertiles in **Table 1**. Among all the participants, 91 (90.1%) were diagnosed as PRETEXT groups I-III and 10 (9.9%) were classified as PRETEXT group IV according to the extent of their liver sections involved on radiographic imaging. While no significant differences were observed in the median age, sex, BMI, birth weight, Scr, AFP and histologic subtypes based on SUA tertiles, the percentage of participants in advanced PRETEXT staging (PRETEXT IV) increased significantly across the SUA tertiles in total HB patients (*p* < 0.01). Among all the annotation factors, participants had a notable upward trend of vascular involvement of the inferior vena cava or hepatic veins (V+) and multifocality (F+) according to SUA tertiles (*p* < 0.05). Univariate analysis of clinical variables confirmed that SUA was positively associated with PRETEXT staging IV (*p* < 0.01) **(** **Supplementary Table S1****)**.

**Table 1:** Patient demographics and clinical characteristics by tertiles of serum uric acid at diagnosis.

Variables	SUA tertiles (mg/dL)			*p*-value
	Tertile 1 (<4.22)	Tertile 2 (4.22-5.68)	Tertile 3 (≥5.68)	
Number of participants ^b^	32 (31.68%)	35 (34.65%)	34 (33.67%)	–
SUA (mg/dL) ^a^	3.38 (2.66-3.89)	4.87 (4.48-5.31)	6.95 (6.31-7.86)	<0.001***
Age (years) ^a^	1.00 (1.00-2.25)	1.00 (1.00-3.00)	1.00 (1.00-2.00)	0.625
Sex ^b^				0.079
Male	25 (78.12%)	20 (57.14%)	18 (52.94%)	
Female	7 (21.88%)	15 (42.86%)	16 (47.06%)	
BMI (kg/m^2^) ^a^	16.35 (15.28-17.35)	16.50 (15.10-17.65)	16.50 (15.33-17.53)	0.413
Birth Weight (kg) ^a^	3.52 (3.20-3.80)	3.50 (3.30-3.60)	3.50 (3.21-3.60)	0.572
SCr (μmol/L) ^a^	20.00 (16.75-25.75)	23.00 (19.00-27.50)	21.00 (19.00-27.00)	0.608
AFP (ng/mL) ^b^				0.308
≤100	0 (0.00%)	0 (0.00%)	1 (2.94%)	
101-1000	1 (3.12%)	1 (2.86%)	1 (2.94%)	
1001–10^6^	16 (50.00%)	11 (31.43%)	8 (23.53%)	
≥10^6^	15 (46.88%)	23 (65.71%)	24 (70.59%)	
Histologic Subtype ^b^				0.053
Epithelial	23 (71.88%)	17 (48.57%)	15 (44.12%)	
Mixed	9 (28.12%)	18 (51.43%)	19 (55.88%)	
PRETEXT ^b^				0.006**
I-III	31 (96.88%)	34 (97.14%)	26 (76.47%)	
IV	1 (3.12%)	1 (2.86%)	8 (23.53%)	
Annotation Factors ^b^				
V-	27 (84.38%)	22 (62.86%)	18 (52.94%)	0.023*
V+	5 (15.62%)	13 (37.14%)	16 (47.06%)	
P-	29 (90.62%)	30 (85.71%)	28 (82.35%)	0.632
P+	3 (9.38%)	5 (14.29%)	6 (17.65%)	
E-	10 (31.25%)	12 (34.29%)	8 (23.53%)	0.604
E+	22 (68.75%)	23 (65.71%)	26 (76.47%)	
F-	28 (87.50%)	33 (94.29%)	25 (73.53%)	0.048*
F+	4 (12.50%)	2 (5.71%)	9 (26.47%)	
R-	26 (81.25%)	31 (88.57%)	32 (94.12%)	0.27
R+	6 (18.75%)	4 (11.43%)	2 (5.88%)	
C-	28 (87.50%)	34 (97.14%)	29 (85.29%)	0.203
C+	4 (12.50%)	1 (2.86%)	5 (14.71%)	
N-	31 (96.88%)	33 (94.29%)	30 (88.24%)	0.443
N+	1 (3.12%)	2 (5.71%)	4 (11.76%)	
M-	26 (81.25%)	30 (85.71%)	25 (73.53%)	0.439
M+	6 (18.75%)	5 (14.29%)	9 (26.47%)	

BMI, body mass index; SCr, serum creatinine; SUA, serum uric acid; AFP, α-fetoprotein; PRETEXT, pretreatment extent of disease.

PRETEXT groups are classified as PRETEXT I-III and PRETEXT IV. Annotation factors are shown as illustrated in Materials and Methods. Epithelial histology subtypes include fetal, embryonal, small-cell undifferentiated, macrotrabecular, cholangioblastic groups. Mixed histology subtypes include teratoid and stromal derivatives groups.

a Values are presented as Median (interquartile range);

b Values are presented as N (%).

*p<0.05, **p<0.01, ***p<0.001.

### 3.2 Association between SUA and PRETEXT staging

**Figure 2:**
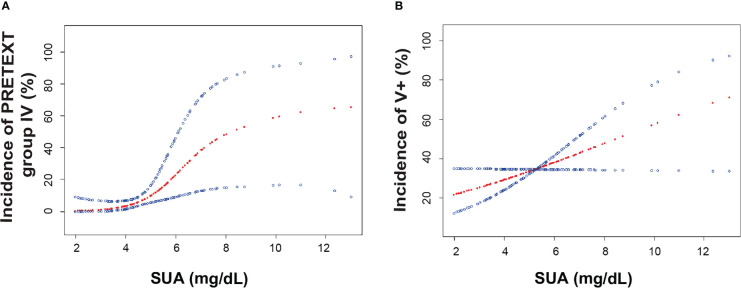
The association of serum uric acid and the incidence of PRETEXT staging IV and vascular involvement. **(A)**. Smoothing spline plots of SUA and the incidence of PRETEXT staging IV in HB patients, adjusted for age, sex, BMI, Scr. **(B)**. Smoothing spline plots of SUA and the incidence of PRETEXT annotation V, adjusted for age, sex, BMI, Scr. Red lines represent the spline plots of SUA concentration (mg/dL) and blue lines represent the 95% confidence intervals of the spline plots. Abbreviations: BMI, body mass index; SCr, serum creatinine; SUA, serum uric acid; AFP, α-fetoprotein; PRETEXT, pretreatment extent of disease.

To further explore the relationship between SUA and PRETEXT staging, we performed binary logistic regression analysis for the occurrence of PRETEXT IV. After adjustment for age, sex, BMI and SCr, SUA levels were positively associated with PRETEXT staging IV **(** **Figure 2A****)**. Subgroup analyses stratified by sex, age and AFP level showed that females, patients aged less than 3 years, and those with AFP≥10^6^ ng/mL had positive associations between SUA and advanced PRETEXT staging (IV). There was no statistically significant interaction of SUA with sex, age and AFP on PRETEXT IV (*p* for interaction > 0.10) **(** **Table 2****)**.

**Table 2:** The association between pretreatment SUA and PRETEXT staging IV stratified by baseline characteristics.

Subgroups	N (%)	OR (95% CI)	*p*-value	*p*-value for interaction
Sex				0.3408
Male Female	63 (62.38%) 38 (37.62%)	1.43 (0.94, 2.17) 1.99 (1.11, 3.58)	0.0941 0.0211*	
Age (years)				0.1701
<3	78 (77.23%)	1.48 (1.08, 2.03)	0.0144*	
≥3	23 (22.77%)	3.01 (0.90, 10.00)	0.0729	
AFP (ng/mL)				0.334
<10^6^	39 (38.61%)	3.22 (0.56, 18.59)	0.1913	
≥10^6^	62 (61.39%)	1.47 (1.09, 2.00)	0.0128*	

*p<0.05.

Furthermore, we implemented multivariate logistic regression analyses to investigate the effect of SUA on PRETEXT staging. Before adjustment for confounding factors, per 1 mg/dL increment of SUA led to an elevated odds for advanced PRETEXT staging (IV) to 1.6 times (OR: 1.60, 95%CI 1.18-2.17, *p*<0.01). We then fully adjusted confounding factors which may affect HB and PRETEXT staging including sex, age, BMI, birth weight, Scr, AFP and histologic subtype. SUA remained positively associated with PRETEXT IV in model I (OR: 1.68, 95%CI 1.19-2.37, *p*<0.01) and model II (OR: 1.72, 95%CI 1.15-2.57, *p*<0.01) respectively **(** **Table 3****).** Hence, our data showed a significant positive relationship between pretreatment SUA and advanced PRETEXT staging (IV).

**Table 3:** The effect of SUA on PRETEXT staging and annotation factors.

Outcomes	Incidence n (%)	Crude		Adjusted model I		Adjusted model II	
		OR (95% CI)	*p*-value	OR (95% CI)	*p*-value	OR (95% CI)	*p*-value
PRETEXT IV ^a^	10 (9.90%)	1.60 (1.18, 2.17)	0.0023**	1.68 (1.19, 2.37)	0.0033**	1.72 (1.15, 2.57)	0.0080**
Annotation Factors ^b^							
V	34 (33.66%)	1.22 (0.99, 1.49)	0.0565	1.27 (1.02, 1.57)	0.0350*	1.39 (1.07, 1.79)	0.0124*
P	14 (13.86%)	1.11 (0.86, 1.43)	0.4097	1.14 (0.87, 1.50)	0.3381	1.26 (0.93, 1.71)	0.1318
E	71 (70.30%)	1.12 (0.90, 1.40)	0.3207	1.13 (0.88, 1.43)	0.3364	1.10 (0.84, 1.43)	0.4910
F	15 (14.85%)	1.23 (0.97, 1.57)	0.0853	1.26 (0.97, 1.64)	0.0848	1.19 (0.89, 1.59)	0.2348
R	12 (11.88%)	0.79 (0.55, 1.13)	0.1979	0.80 (0.55, 1.17)	0.2551	0.71 (0.47, 1.09)	0.1170
C	10 (9.90%)	1.13 (0.84, 1.52)	0.4133	1.13 (0.84, 1.52)	0.4246	1.30 (0.92, 1.85)	0.1393
N	7 (6.93%)	1.33 (0.97, 1.83)	0.0746	1.39 (0.98, 1.98)	0.0685	1.98 (1.04, 3.78)	0.0579
M	20 (19.80%)	1.04 (0.83, 1.31)	0.7387	1.08 (0.83, 1.39)	0.5693	1.07 (0.80, 1.43)	0.6285

a PRETEXT staging is grouped as PRETEXT I-III or PRETEXT IV.

b Annotation factors are classified as present or absent in each category.

Adjusted model I: Adjusted for age, sex, BMI, SCr. Adjusted model II: Adjusted for age, sex, BMI, SCr, birth weight, histologic subtype, AFP.

*p<0.05, **p<0.01.

### 3.3 Association between SUA and annotation factors

We then examined whether SUA had an effect on the various PRETEXT annotation factors in HB patients. Smoothing spline plots of SUA and annotations showed that as SUA concentrations raised, patients presented higher chance of tumor invasion in the inferior vena cava and hepatic veins (V+) **(** **Figure 2B****)**. When SUA was considered as a continuous variable, for each increment of SUA, multivariate adjusted model yielded 1.27 times of odds for V+ involvement in model I (OR: 1.27, 95% CI 1.02-1.57, *p*<0.05) and 1.39 times of odds in model II (OR: 1.39, 95% CI 1.07-1.79, *p*<0.05) **(** **Table 3****)**. Similarly, the positive association was observed across SUA tertiles with the lowest tertile as the reference **(** **Supplementary Table S2****)**. We demonstrated that the highest SUA tertile were positively associated with the presence of annotation factor V+ in both the crude and adjusted models **(** **Supplementary Table S2****)**. In addition to that, the odds ratio for portal venous involvement (P+) suggested SUA could have a positive effect on P+ involvement, however, regression models didn’t achieve statistical significance **(** **Table 3****)** probably due to the small number of P+ cases.

Accordingly, we set up another group named “vascular invasion (V+/P+)” by aggregating V+ or/and P+ cases together **(** **Table 4****)**. This group specified patients with at least one of the two factors present. As a continuous variable, SUA was proved to be associated with vascular invasion (V+/P+) in adjusted model II (OR: 1.29, 95% CI 1.01-1.64, *p*<0.05). Likewise, the positive relationship with vascular invasion was demonstrated in crude and adjusted models across SUA tertiles **(** **Table 4****)**. Together, these data further confirmed that pretreatment SUA were positively associated with vessel involvement in HB patients.

**Table 4:** The association of pretreatment SUA concentrations with vascular invasion (V+/P+).

SUA (mg/dL)	Crude		Adjusted model I		Adjusted model II	
	OR (95% CI)	*p*-value	OR (95% CI)	*p*-value	OR (95% CI)	*p*-value
Continuous	1.18 (0.97, 1.44)	0.1033	1.20 (0.97, 1.47)	0.0957	1.29 (1.01, 1.64)	0.0396*
Tertiles						
Tertile 1(<4.22)	Reference		Reference		Reference	
Tertile 2(4.22-5.68)	2.38 (0.81, 6.99)	0.1144	2.80 (0.85, 9.23)	0.0902	4.50 (1.13, 17.84)	0.0324*
Tertile 3(≥5.68)	3.57 (1.22, 10.46)	0.0202*	4.42 (1.38, 14.16)	0.0124*	8.20 (2.03, 33.13)	0.0031**

Adjusted model I: Adjusted for age, sex, BMI, SCr.

Adjusted model II: Adjusted for age, sex, BMI, SCr, birth weight, histologic subtype, AFP.

* *p*<0.05, ** *p*<0.01.

We also investigated other PRETEXT annotation factors. Although odds ratios in the adjusted models showed SUA might have a positive effect on E+, F+, C+, N+, M+, and an adverse effect on R+ respectively, statistical significance were not achieved **(** **Table 3****)**.

### 3.4 Posttreatment SUA and POSTTEXT staging

**Figure 3:**
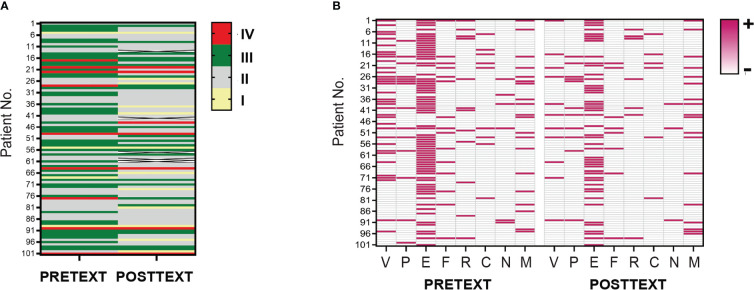
PRETEXT and POSTTEXT assessment in patient cohort of HB. **(A)**. Tumor extent before and after neoadjuvant chemotherapy in the full cohort of patients were evaluated by PRETEXT and POSTTEXT staging. Each row in the heatmap demonstrates one patient. Yellow, grey, green and red lines indicate stage I, II, III, IV. **(B)**. Distribution of radiological annotation factors in patient cohort before and after chemotherapy are shown. Pink and grey lines in the map indicate presence and absence of the annotation factors, respectively. PRETEXT, pretreatment extent of disease; POSTTEXT, posttreatment extent of disease.

Since we had demonstrated pretreatment SUA were associated with advanced PRETEXT staging and vascular invasion, we further investigated whether SUA levels changed with the treatment, and how this reflected patients’ response to the therapy. Hence, we then examined SUA concentrations and evaluated radiologic imaging of patients at 2 or 4 cycles of NAC. Among 101 HB participants, 96 patients had POSTTEXT imaging and laboratory data collected and analyzed. Thirty-nine tumors (40.63%) were down-staged for POSTTEXT after 4 cycles of chemotherapy(*p*<0.05). Of 34 patients who had V+ involvement at diagnosis, 24(70.59%) were V- following chemotherapy. Nine out of 14 patients(64.29%) who had P+ involvement were down-staged(*p*<0.01). PRETEXT/POSTTEXT staging and annotation factors of the full cohort were shown in **Figure 3**.

Posttreatment serum AFP concentrations were reduced to 11041ng/mL(657-35350ng/mL). The rate of change in AFP levels upon treatment was 84.98%(17.59-96.74%) which reflected tumor response to 4 cycles of NAC before surgery. SUA levels after chemotherapy (4.18 mg/dL, 3.43-5.01mg/dL) were significantly decreased compared to those at diagnosis (4.99mg/dL, 3.94-6.33mg/dL) (*p*<0.01).

We then explored the association between posttreatment SUA and POSTTEXT staging and vascular involvement. Logistic regression models showed SUA following NAC were positively associated with advanced POSTTEXT(III/IV) staging **(** **Table 5**, **Supplementary Table S3****)**. While vascular invasion were down-staged in most of the participants after chemotherapy, SUA still exhibited significant relationship with V+/P+ involvement **(** **Table 5****)**.

**Table 5:** The evaluation of SUA after neoadjuvant therapy with POSTTEXT staging and vessel involvement.

SUA (mg/dL)	Crude		Adjusted model I		Adjusted model II	
	OR (95% CI)	*p*-value	OR (95% CI)	*p*-value	OR (95% CI)	*p*-value
POSTTEXT III/IV	1.76 (1.18, 2.64)	0.0057**	2.04 (1.28, 3.24)	0.0026**	2.79 (1.48, 5.24)	0.0014**
V+/P+	6.32 (2.76, 14.49)	0.0001***	8.82 (2.96, 26.26)	0.0001***	13.60 (2.88, 64.31)	0.0010***

Adjusted model I: Adjusted for age, sex, BMI, SCr.

Adjusted model II: Adjusted for age, sex, BMI, SCr, birth weight, histologic subtype, AFP.

**p<0.01, ***p<0.001

### 3.5 SUA and patient outcomes

We also analyzed the effect of post-NAC SUA levels on patient survivals. After a median (range) follow-up of 67 months (1–190 months), there were 8 events of relapse and 9 events of progression of disease. Among them, 9 patients had pulmonary metastases. Numbers of patients who had relapse across SUA tertiles 1/2/3 were 1(10%)/4(40%)/5(50%). Those who had progression across SUA tertiles were 0 (0.0%)/2(28.57%)/5(71.43%). Those who developed lung metastasis across SUA tertiles were 0(0.0%)/3(33.33%)/6(66.67%). Patients with the highest tertile of posttreatment SUA had elevated frequency of relapse/progression events.

**Figure 4:**
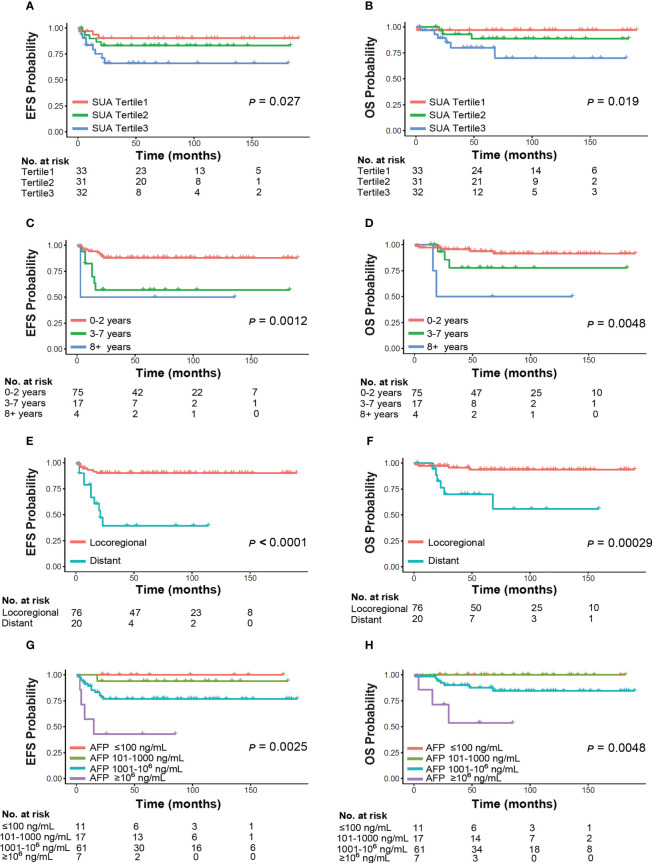
Event-free survival and overall survival by clinical characteristics of HB. Panels demonstrate subgroup analyses by **(A, B)** SUA, **(C, D)** age, **(E, F)** metastases status, **(G, H)** AFP. SUA and AFP levels were examined post-neoadjuvant chemotherapy. Abbreviations: SUA, serum uric acid; AFP, α-fetoprotein.

Among all the subjects, 10 (9.90%) patients died eventually. The overall 5-year EFS was 80.50%, and the overall 5-year OS was 88.90%, respectively. Survival was significantly different across post-NAC SUA tertiles (*p*<0.05), with the highest SUA tertile had worse 5-year EFS and OS rates **(** **Figure 4****)**. The 5-year EFS by SUA tertiles were 90.30%, 83.30%, 66.20%. The 5-year OS by SUA tertiles were 97.00%, 88.70%, 80.00%, respectively. Meanwhile, significant differences of survival in our cohort existed by other factors including age at diagnosis, metastases status, and post-NAC AFP levels **(** **Figure 4****)**. Those patients diagnosed above 8 years old, presented with distant tumor stage and AFP remained ≥10^6^ ng/mL after NAC had worse survival outcomes (*p*<0.01).

## 4 Discussion

Multiple studies have shown that hyperuricemia has various effects on the cancer risks and prognosis (23–25). In this study, we demonstrated that the percentages of HB patients with advanced PRETEXT staging (PRETEXT IV) and vascular invasion at diagnosis increased significantly across the SUA tertiles. Moreover, after adjustment for confounding factors, pretreatment SUA level was positively associated with risk of advanced PRETEXT staging (IV) and vascular invasion (V+/P+). In addition, following 2 or 4 cycles of NAC, SUA level was significantly decreased in response to the chemotherapy, and remained positively associated with advanced POSTTEXT staging (III/IV) and vessel involvement. Finally, patients’ survival outcomes were significantly different across posttreatment SUA tertiles, with the highest tertile exhibited worse 5-year EFS and OS rates. To our knowledge, this is the first study that investigate the relationship of elevated SUA level with pediatric HB staging and extra-parenchymal tumor features.

Consistent with what we found in pediatric HB patients; emerging evidence of epidemiological studies have linked SUA to liver cancer in adulthood. A retrospective analysis of 288 advanced HCC patients revealed that SUA were negatively correlated with the survival time (15). This was also evidenced by other research showing HCC patients with high pre-operative SUA had worse prognosis after surgery (26). Another study reported high SUA level was a risk factor of HCC recurrences (27).

Our study indicated that increment of SUA level was associated with increased odds of advanced PRETEXT/POSTTEXT staging, after fully adjustment for confounding factors. PRETEXT sating IV, compared to other PRETEXT groups (I, II, III), defines the greatest extent of liver sections invaded by tumor, and has evidenced by previous studies as a poor prognostic factor (5, 6). Similar to our results, other research supported adult HCC patients at TNM III+IV stages showed higher SUA level than those at I+II stages and healthy subjects (28). Also, pre-operative SUA was found to be associated with TNM stages in esophageal squamous cell carcinoma (29).

Apart from assessment of tumor extension, the annotation factors (V, P, E, F, R, C, N, M) denote the extra-parenchymal tumor features which were refined in PRETEXT system for standardization of disease evaluation (6). Our study demonstrated that SUA was independently related to vascular invasion (V+/P+) at diagnosis and following NAC. Whereas the highest SUA tertile were associated with the presence of hepatic venous/inferior vena cava involvement (V+) at diagnosis, the relationship of SUA with portal venous involvement (P+) didn’t achieve statistical significance. One explanation to this discrepancy was that the number of P+ cases was insufficient to test the effect of SUA on portal venous involvement. In addition, gradation of vessel invasion, which reflected number and degree of the vessels involved, could be different among V+ and P+ participants.

The latest updated PRETEXT staging system has been proved to be the primary method of risk stratification for HB in numerous clinical trials and real-world setting (30–33). Children with advanced PRETEXT staging and vessel involvement should be treated with NAC at diagnosis before surgical resection can be performed (5). Accordingly, POSTTEXT staging after 2 or 4 cycles of NAC further assesses tumor response to the chemotherapy and is crucial to evaluate tumor resectability (34, 35). Patients with advanced POSTEXT staging and unresectable vessel involvement are candidates for liver transplantation (34, 36).

Therefore, our study uncovered the effect of pretreatment SUA in disease evaluation at diagnosis, most importantly, we displayed that SUA level decreased in response to the downstaging of tumor extent and vessel involvement following NAC. High SUA after chemotherapy reflected poor tumor responses to the NAC. This made it feasible for monitoring the management of HB. In our cohort, patients with the highest tertile of posttreatment SUA were prone to relapse or progression of the disease. Those patients exhibited worse 5-year EFS and OS rate. These results suggested SUA provided prognostic information for patient outcomes.

Whereas radiological parameters in PRETEXT/POSTTEXT system have proved to be prognostic for patient outcomes (37–39), there aren’t much option for laboratory biomarkers. AFP is currently the only serum biomarker of HB that universally utilized for diagnosis and outcome prediction (39, 40). AFP contributes to the backbone groups of global risk stratification system of HB (7). In our study, we showed patients with AFP≥10^6^ ng/mL after NAC had worse survival outcomes. This was consistent with previous reports that the reduction of AFP following NAC, together with tumor shrinkage rate, were significantly related to the risk of tumor recurrence (36).

Given to the association of SUA with PRETEXT/POSTTEXT staging and vascular invasion, it could be used as a complementary tool with AFP for disease evaluation and surveillance. High levels of SUA may be conducive to identifying patients with potential poor prognosis and facilitating treatment selection.

It is worth mention that the elevated SUA levels discussed in this study were not caused by TLS (41). Spontaneous laboratory TLS was ruled out in our study by exclusion criteria. Factors such as age at diagnosis, sex, initial AFP level, pathologic subtype, low birth weight, that had been reported to affect the risk and prognosis of HB (5, 7, 30, 31, 42) were all included in the confounding factors for adjustment. Additionally, BMI and SCr were also adjusted since body fat and kidney function were related to SUA level (17, 18, 43, 44). Hence, the association of SUA levels with PRETEXT/POSTTEXT staging and annotations were independent of all these confounders.

The underlying mechanism of how uric acid participate in cancer development has not been fully elucidated. Uric acid is the end product of the purine metabolism pathway, which produces reactive oxygen species (ROS) as by-products. ROS plays a key role in cancer development by promoting oxidative DNA damage, and contributing to mutations in tumor-suppressing genes (45, 46). Wu et al. (15) found uric acid and other purine metabolites were significantly upregulated in the serum and liver samples of HCC rats. Inhibiting uric acid production attenuated liver ROS production, thus significantly delayed the progression of liver cancer (15). Similar to these results, Springer et al. confirmed that a large amount of ROS was generated while uric acid was produced in the condition of cancer (47). In addition, increased oxidative reactions may activate transcription factors that promote cell proliferation and migration, leading to tumor progression (48). These evidences imply that SUA contributes to the carcinogenesis.

Moreover, recent studies have indicated a new role of uric acid in the Wnt/β-catenin signaling pathway. Activation of Wnt/β-catenin pathway occurs in up to 80% of HB, and is believed to play a critical role in the pathogenesis of HB (49, 50). Translocation of β-catenin to the nucleus drives tumorigenesis by deregulating downstream oncogenes (51–53). Recent study showed the role of uric acid in the Wnt/β-catenin signaling in hyperuricemic nephropathy (HN). Increased accumulation of uric acid in renal tubular epithelial led to inflammation and fibrosis *via* Wnt/β-catenin pathway, and could be attenuated by Apigenin (API) (54, 55). API has been well reported of its anticancer effect closely related to the Wnt/β-catenin pathway (56). Whether uric acid may affect HB tumorigenesis *via* Wnt/β-catenin pathway prompts further research.

There are several limitations in our study. First, the current research was a retrospective study performed in single institution, and a significant number of patients were excluded due to the missing clinical information. This had inherent potential for selection bias. Second, the sample size of our study was relatively small, which was a common drawback for many other studies of rare tumors with low annual incidence. Some subgroups had very few positive events. Accordingly, subgroup analyses were hard to achieve statistical significance. Third, disparities in treatment regimen and surgery strategy were not included in the analyses. Last, all of the participants fit into the inclusion criteria of this study were aged under 10 years, making it impossible to investigate the role of SUA in HB after puberty. This was also due to the fact that most HB patients present before 5 years of age (1). Given that SUA levels increase following hormonal changes after puberty, gender-specific reference intervals need to be examined in those patients in the future studies.

Nonetheless, a major advantage is that this study fills the current gaps in literature by linking SUA, a simple and easily performed laboratory test, to the updated PRETEXT/POSTTEXT staging system which provides crucial evaluation of disease and defines treatment guidelines. We analyzed SUA as both continuous and classified variables to enhance the robustness of the results. Additionally, we included other risk factors of poor prognosis such as age, birth weight, AFP and histologic subtypes, as well as factors affecting SUA levels including BMI and SCr as confounders in this study. Therefore, the positive association of SUA and PRETEXT/POSTTEXT staging was independent of these risk factors.

## 5 Conclusion

Elevated SUA is associated with a high occurrence of advanced PRETEXT/POSTTEXT staging and vascular invasion at diagnosis and following NAC in HB patients. High posttreatment SUA reflects poor tumor responses to NAC. This is meaningful in the clinical setting for disease assessment and treatment selection. Further research on larger and longitudinal cohort studies are warranted to assess the alterations of SUA after surgery.

## Data availability statement

The original contributions presented in the study are included in the article/**Supplementary Material**. Further inquiries can be directed to the corresponding authors.

## Ethics statement

The studies involving human participants were reviewed and approved by Ethics Committee of Xinhua Hospital. Written informed consent to participate in this study was provided by the participants’ legal guardian/next of kin.

## Author contributions

YZ: Conceptualization, Methodology, Software, Investigation, Validation, Data Curation, Writing - Original Draft. JL: Conceptualization, Methodology, Investigation. YM: Software, Investigation. MT: Validation. XY: Conceptualization, Writing - Review & Editing, Supervision. LS: Conceptualization, Writing - Review & Editing, Supervision. All authors have approved the manuscript.
