# Supplementary material for: Elevated serum uric acid is associated with the risk of advanced staging and vascular involvement in patients with hepatoblastoma: a 14-year retrospective study
Source: Front Oncol. 2023 Apr 14;13:1144349. doi: 10.3389/fonc.2023.1144349 (PMC10140562; doi:10.3389/fonc.2023.1144349)
Supplement: Supplementary file 1 [file Table_1.docx]

Supplementary Material

**Supplementary Table S1. Univariate analysis of clinical variables for PRETEXT staging**

| **Variables** | **PRETEXT IV** | | **V** | | **F** | |
| --- | --- | --- | --- | --- | --- | --- |
|  | **OR (95% CI)** | ***p*-value** | **OR (95% CI)** | ***p*-value** | **OR (95% CI)** | ***p*-value** |
| Age (years) | 1.14 (0.88, 1.49) | 0.3163 | 1.16 (0.96, 1.40) | 0.1204 | 1.32 (1.07, 1.64) | 0.0095** |
| Sex |  |  |  |  |  |  |
| Male | Reference |  | Reference |  | Reference |  |
| Female | 3.75 (0.88, 16.00) | 0.0741 | 0.66 (0.28, 1.57) | 0.3506 | 1.55 (0.51, 4.69) | 0.4356 |
| BMI (kg/m^2^) | 0.92 (0.67, 1.27) | 0.6146 | 0.99 (0.85, 1.15) | 0.8628 | 0.85 (0.64, 1.14) | 0.2746 |
| Birth Weight (kg) | 0.88 (0.22, 3.57) | 0.8560 | 0.95 (0.41, 2.22) | 0.9116 | 0.48 (0.16, 1.48) | 0.2039 |
| SCr (μmol/L) | 1.03 (0.97, 1.09) | 0.3866 | 1.02 (0.96, 1.05) | 0.8244 | 1.03 (0.98, 1.08) | 0.2427 |
| SUA (mg/dL) | 1.60 (1.18, 2.17) | 0.0023** | 1.22 (0.99, 1.49) | 0.0565 | 1.23 (0.97, 1.57) | 0.0853 |

** ***p*＜0.01**

**Supplementary Table S2. Pretreatment tumor involvement of inferior vena cava or hepatic veins (V+) across tertiles of SUA**

| **SUA (mg/dL)** | **Crude** | | **Adjusted model I** | | **Adjusted model II** | |
| --- | --- | --- | --- | --- | --- | --- |
|  | **OR (95% CI)** | ***p*-value** | **OR (95% CI)** | ***p*-value** | **OR (95% CI)** | ***p*-value** |
| Tertile 1 (＜4.22) | Reference |  | Reference |  | Reference |  |
| Tertile 2 (4.22-5.68) | 2.56 (0.83, 7.86) | 0.1004 | 3.38 (0.98, 11.61) | 0.0530 | 6.68 (1.49, 29.92) | 0.0131* |
| Tertile 3 (≥5.68) | 3.85 (1.26, 11.74) | 0.0177* | 5.25 (1.57, 17.54) | 0.0070** | 11.97 (2.58, 55.51) | 0.0015** |

Adjusted model I: Adjusted for age, sex, BMI, SCr.

Adjusted model II: Adjusted for age, sex, BMI, SCr, birth weight, histologic subtype, AFP.

* ***p*＜0.05,** ** ***p*＜0.01**

**Supplementary Table S3. The association of posttreatment SUA tertiles with POSTTEXT staging III / IV**

| **SUA (mg/dL)** | **Crude** | | **Adjusted model I** | | **Adjusted model II** | |
| --- | --- | --- | --- | --- | --- | --- |
|  | **OR (95% CI)** | ***p*-value** | **OR (95% CI)** | ***p*-value** | **OR (95% CI)** | ***p*-value** |
| Tertile 1(＜3.65) | Reference |  | Reference |  | Reference |  |
| Tertile 2(3.65-4.66) | 1.52 (0.49, 4.75) | 0.4717 | 1.59 (0.49, 5.15) | 0.4370 | 2.02 (0.57, 7.17) | 0.2773 |
| Tertile 3(≥4.66) | 2.89 (0.97, 8.58) | 0.0561 | 4.07 (1.21, 13.70) | 0.0236* | 5.79 (1.37, 24.39) | 0.0168* |

Adjusted model I: Adjusted for age, sex, BMI, SCr.

Adjusted model II: Adjusted for age, sex, BMI, SCr, birth weight, histologic subtype, AFP.

* ***p*＜0.05**
